# The ubiquitin ligase TRIM27 functions as a host restriction factor antagonized by *Mycobacterium tuberculosis* PtpA during mycobacterial infection

**DOI:** 10.1038/srep34827

**Published:** 2016-10-04

**Authors:** Jing Wang, Jade L. L. Teng, Dongdong Zhao, Pupu Ge, Bingxi Li, Patrick C. Y. Woo, Cui Hua Liu

**Affiliations:** 1 CAS key Laboratory of Pathogenic Microbiology and Immunology, Institute of Microbiology, Chinese Academy of Sciences, Beijing 100101, China; 2 Department of Microbiology, The University of Hong Kong, Hong Kong, China; 3 Savaid Medical School, University of Chinese Academy of Sciences, Beijing 101408, China

## Abstract

Macrophage-mediated innate immune responses play crucial roles in host defense against pathogens. Recent years have seen an explosion of host proteins that act as restriction factors blocking viral replication in infected cells. However, the essential factors restricting *Mycobacterium tuberculosis* (Mtb) and their regulatory roles during mycobacterial infection remain largely unknown. We previously reported that Mtb tyrosine phosphatase PtpA, a secreted effector protein required for intracellular survival of Mtb, inhibits innate immunity by co-opting the host ubiquitin system. Here, we identified a new PtpA-interacting host protein TRIM27, which is reported to possess a conserved RING domain and usually acts as an E3 ubiquitin ligase that interferes with various cellular processes. We further demonstrated that TRIM27 restricts survival of mycobacteria in macrophages by promoting innate immune responses and cell apoptosis. Interestingly, Mtb PtpA could antagonize TRIM27-promoted JNK/p38 MAPK pathway activation and cell apoptosis through competitively binding to the RING domain of TRIM27. TRIM27 probably works as a potential restriction factor for Mtb and its function is counteracted by Mtb effector proteins such as PtpA. Our study suggests a potential tuberculosis treatment via targeting of the TRIM27-PtpA interfaces.

The innate immunity is a universal and ancient form of host defense system against invading pathogens. During infection, a variety of receptors on/in macrophages called pattern recognition receptor (PRR) can recognize conserved products of pathogens (pathogen-associated molecular patterns, PAMP) to stimulate certain host immune and inflammatory signaling pathways upon pathogen infection. Central to the innate immune signaling, the nuclear factor-κB (NF-κB) and JNK/p38 mitogen-activated protein kinase (MAPK) pathways are crucial in regulating cytokine production^1^,^2^ downstream of recognition of PAMPs.

Upon infection by pathogens, some host proteins, called restriction factors^3^, are stimulated to enhance host innate immune responses to suppress the intracellular survival of pathogens. In recent years, there has been an explosion in the investigation of host proteins that act as restriction factors to block the replication cycles of viruses^4^,^5^,^6^,^7^,^8^,^9^. The tripartite motif containing (TRIM) proteins are involved in a broad range of biological processes including cell proliferation, differentiation, development and apoptosis. The majority of TRIM family members possess a conserved RBCC motif, which consists of a RING domain and one or two B-Box domains followed by a coiled-coil region. Previous reports have shown that the TRIM protein family is emerging as a key component of host antiviral innate immunity^10^. Beginning with the identification of TRIM5α as a post-entry restriction factor against retroviruses, a lot of progress has been made on how TRIM proteins influence immunity over the past decade^11^,^12^, including the identification of TRIM56, which dictates antiviral restriction of influenza A and B viruses by impeding viral RNA synthesis^13^,^14^.

In order to promote virulence and evade detection and immune clearance, bacterial pathogens have evolved various effector proteins to perturb host immune responses by targeting the key components of innate immune system. For example, *Salmonella typhimurium* expresses the kinase SteC, which phosphorylates MKK1/2, leading to activation of ERK1/2 and reorganization of the actin cytoskeleton, which restrains bacterial growth^15^; and *Legionella pneumophila* translocates LegK1 into macrophages to activate NF-κB signaling pathway, which causes the inhibition of cell apoptosis and promotes the intracellular survival of bacteria^2^. Understanding the elaborate strategies that pathogens employ to subvert their host immune responses is helpful to identify novel approaches for prevention and treatment of infection.

*Mycobacterium tuberculosis* (Mtb), the causative agent of tuberculosis (TB), is one of the most dangerous infectious pathogens worldwide. In 2014, more than 9 million people were estimated as new TB cases and about 1.5 million died from the disease^15^. As an intracellular pathogen, Mtb secretes a number of proteins into host macrophages to interfere with various cellular processes, such as apoptosis, autophagy and the signaling pathways^13^,^16^,^17^,^18^. Mtb PtpA is a secreted low-molecular weight tyrosine phosphatase required for intracellular survival of mycobacteria, and it also acts as a pivotal modulator of host innate immune responses^14^,^18^,^19^ and cell apoptosis^20^. We previously revealed that PtpA inhibits the innate immune signaling pathways and phagosome maturation by co-opting host ubiquitin via a previously unknown ubiquitin-interacting motif-like (UIML) region, which leads to the suppression of innate immunity^16^. In this work, we identified TRIM27 as a novel PtpA-interacting host protein. TRIM27 has been previously characterized as a transcription repressor through interaction with retinoblastoma-associate protein (Rb), Enhancer of the Polycomb 1 (EPC1), or Mi-2b-containing histone deacetylase complex in the nucleus^21^,^22^,^23^,^24^. Harboring a RING domain, TRIM27 has also been shown to be an E3 ubiquitin ligase^25^,^26^,^27^,^28^,^29^ and to possess SUMO E3 ligase activity as well^30^. We further demonstrated that TRIM27 restricts the intracellular survival of mycobacteria, suggesting that it is a potential host restriction factor for Mtb. Moreover, we showed that TRIM27 suppresses the intracellular survival of mycobacteria by enhancing host immune-inflammatory responses mediated by JNK/p38 pathways as well as cell apoptosis. Interestingly, Mtb PtpA antagonizes TRIM27-promoted JNK/p38 MAPK pathway activation and cell apoptosis through competitively binding to the RING domain of TRIM27. Our findings highlight an evolutionary dynamics of interactions between a host restriction factor and its pathogen antagonist during mycobacterial infection.

## Results

### Identification of Mtb PtpA interacting host proteins

**Figure 1:**
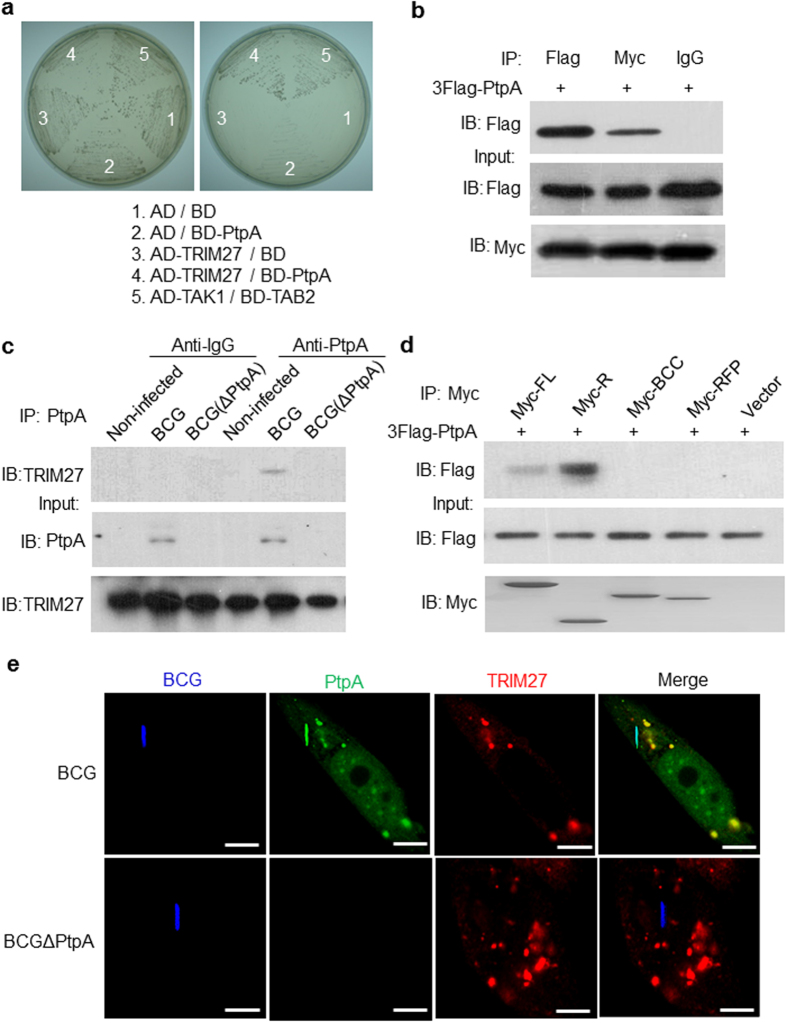
Identification of TRIM27 as a *Mycobacterium tuberculosis* (Mtb) PtpA-interacting host protein. (**a**) Mtb PtpA interacts with TRIM27 in the yeast two-hybrid assay. Yeast strains were transformed with indicated vectors in which TAK1 and TAB2 interaction served as a positive control. Left, high-stringency. Right, low-stringency. (**b**) Immunoblot analysis (IB) of proteins immunoprecipitated (IP) with immunoglobulin G (IgG; control), anti-Flag or anti-Myc from lysates of HEK293T cells transfected with vectors encoding Flag-tagged PtpA and Myc-tagged TRIM27 (top), and immunoblot analysis without immunoprecipitation (Input; below). (**c**) Immunoblot analysis of proteins immunoprecipitated with anti-PtpA from lysates of U937 cells infected with wild-type (WT) BCG or PtpA deleted BCG (BCGΔPtpA) for 24 h. Non-infected cells were used as a control. (**d**) Immunoblot analysis of proteins immunoprecipitated with anti-Myc from lysates of HEK293T cells transfected with vectors encoding Flag-tagged PtpA and Myc-tagged full-length (FL) TRIM27 or its truncated forms (R, RING; BCC, B-Box region and coiled-coil region; RFP, RFP region). (**e**) Confocal microscopy of U937 cells infected with BCG at a MOI of 20. Mtb PtpA (Green) colocalizes with TRIM27 (Red) in U937 cells. Bacteria (Blue) were stained with Alexa 350 carboxylic acid succinimidyl eater. Scale bars, 10 μm.

In our efforts to conduct more in-depth investigation into the immune-regulatory mechanisms of Mtb PtpA, we identified another novel Mtb PtpA-interacting host protein TRIM27 through a yeast two-hybrid assay from a mouse cDNA library (Fig. 1a). Among all the candidate Mtb PtpA-interacting host proteins we identified, TRIM27 aroused our interest because many members of TRIM family have been shown to play crucial role in the process of pathogen defense^10^,^11^,^12^. We confirmed the interactions between Mtb PtpA and TRIM27 by co-immunoprecipitation in HEK293T cells cotransfected with vectors encoding PtpA and TRIM27 (Fig. 1b) and in U937 cells infected with BCG strains (Fig. 1c). We further showed that Mtb PtpA interacts with TRIM27 via the RING domain (Fig. 1d), a region defining the E3 ubiquitin ligase activity of TRIM27. Deletion of RING domain disrupts the interactions between TRIM27 and PtpA (Supplementary Fig. 1). In addition, immunouorescence confocal microscopy analysis data indicated that the secreted protein PtpA, but not the bacterium itself, co-localized with TRIM27 in macrophage cells during mycobacterial infection (Fig. 1e). These results suggest that the Mtb PtpA-interacting TRIM27 may play an important regulatory role during mycobacterial infection dependent on its E3 ubiquitin ligase activity, which could be potentially counteracted by the Mtb effector protein PtpA.

### TRIM27 restricts the survival of mycobacteria in macrophages

**Figure 2:**
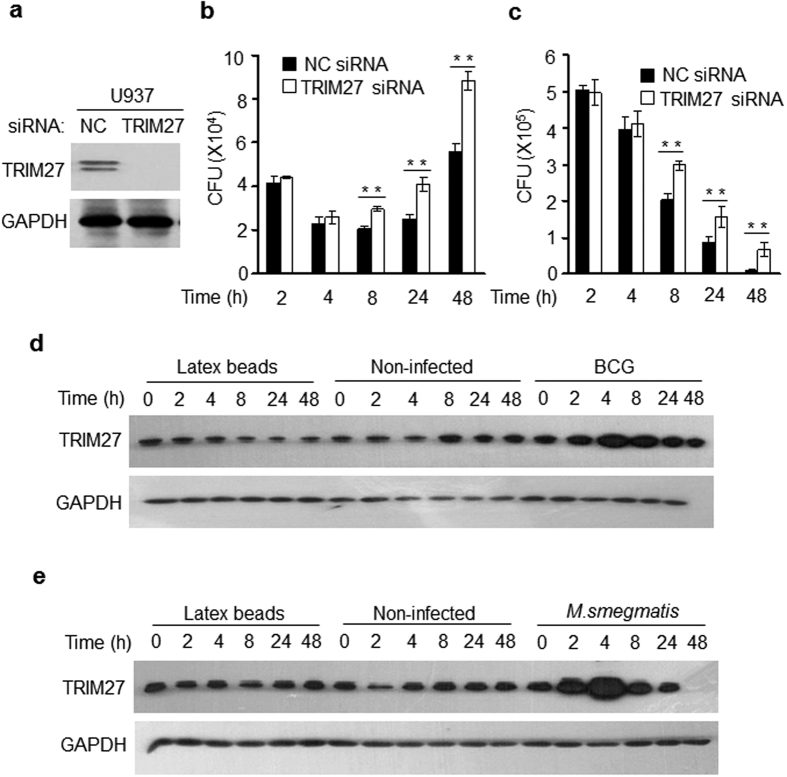
TRIM27 restricts the intracellular survival of mycobacteria. (**a**) The protein levels of TRIM27 were analyzed by immunoblot analysis in U937 cells transfected with luciferase siRNA (NC) or TRIM27 siRNA. (**b,c**) Survival of BCG (**b**) and *M. smegmatis* (**c**) in U937 cells infected with BCG or *M. smegmatis* at a MOI of 10 for 0–48 h. Non-infected cells were used as a control. (**d,e**) The levels of TRIM27 were examined in U937 cells infected with BCG (**d**) or *M. smegmatis* (**e**) at a MOI of 10 for 0–24 h. Non-infected cells and cells treated with latex beads served as control groups. Data are shown as the means ± s.e.m. *P < 0.05 and **P < 0.01.

To better understand the physiological role of TRIM27 during mycobacterial infection, we then sought to examine whether TRIM27 regulates the intercellular survival of BCG or *M. smegmatis*, the widely used model organisms used for elucidating the pathogenesis of mycobacteria in macrophages. Macrophage-like U937 cells (differentiated from U937 human monocytic cells) were transfected with negative control small interfering RNA (NC siRNA) or TRIM27-specific siRNA, followed by infection with mycobacteria. Immunoblot analysis was performed to test the knockdown efficiency of siRNAs (Fig. 2a). The mycobacterial colony-forming units (CFUs) decreased from 2 h after infection of either BCG or *M. smegmatis*. Knock-down of TRIM27 significantly promoted the intercellular survival of BCG (Fig. 2b) and *M. smegmatis* (Fig. 2c) from 8 h post-infection of U937 cells. Different from *M. smegmati*s of which the CFUs decreased consistently, the CFUs of BCG increased after 24 h post-infection. In addition, infection of BCG (Fig. 2d) or *M. smegmatis* (Fig. 2e) induced expression of TRIM27 in U937 cells at 2 h post-infection, followed by decline at later time points. Controls with non-infected U937 cells and with latex beads at each time point were used to demonstrate the specificity of TRIM27 expression in response to mycobacterial infection. As a whole, these results indicate the possibility that TRIM27 might function as a host restriction factor being stimulated upon the invasion of mycobacteria to suppress the intracellular survival of the pathogens.

### TRIM27 promotes JNK and p38 pathway activation and suppresses NF-κB activation

**Figure 3:**
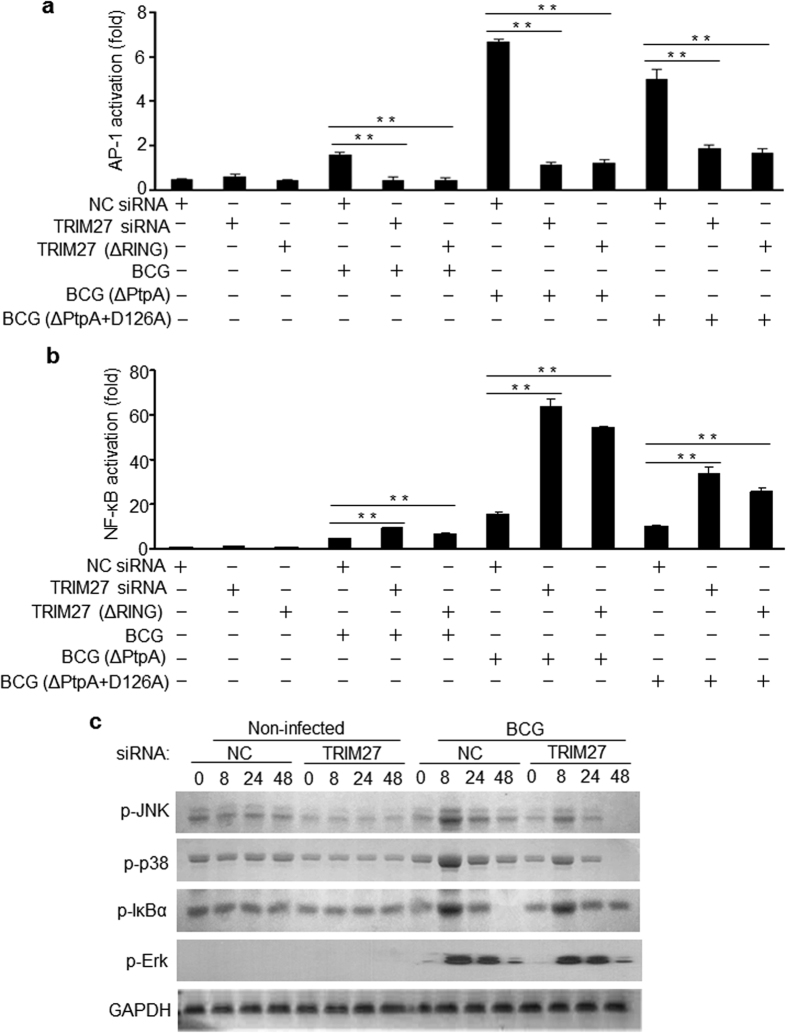
TRIM27 promotes the activation of JNK and p38 pathways and suppresses NF-κB activation. (**a,b**) Luciferase assay of of AP-1 **(a**) and NF-κB (**b**) activation in U937 cells. Cells were transfected with luciferase siRNA (NC siRNA) or TRIM27 siRNA or TRIM27 siRNA complemented with TRIM27 (ΔRING), and then were infected with WT BCG or BCG (ΔPtpA) or the BCG (ΔPtpA) complemented with PtpA D126A (ΔPtpA+D126A) at a MOI of 10 for 24 h. (**c**) Immunoblot analysis of phosphorylated IκBα, JNK, p38, Erk and GAPDH (loading control) in U937 cells transfected with luciferase siRNA (NC siRNA) or TRIM27 siRNA and infected with BCG at a MOI of 10 for 0–48 h. Non-infected cells served as a control group. Data are shown as the means ± s.e.m. *P < 0.05 and **P < 0.01.

Central to the innate immune system, the NF-κB and JNK/p38 signaling pathways control inflammatory cytokine induction to defend against invading bacteria. To investigate the underlying mechanisms by which TRIM27 modulates innate immune responses, dual-luciferase reporter assay and immunoblot analysis were performed to examine the effects of TRIM27 on immune signaling pathways. The data revealed that knockdown of TRIM27 in U937 macrophage cells efficiently blocked JNK/p38 signal pathway activation induced by BCG infection (Fig. 3a), whereas it promoted the BCG-stimulated NF-κB activation in a RING domain-dependent manner (Fig. 3b). Consistently, overexpression of TRIM27 in HEK293T cells induced a significant activation of JNK/p38 MAPK pathways stimulated by the constitutively active Rac mutant RacL61 and partially inhibited TNF-induced activation of NF-κB pathway (Supplementary Fig. 2a,b), while had no effects on Erk pathway activation (Supplementary Fig. 2c). Thus, TRIM27 promotes JNK and p38 pathway activation and suppresses NF-κB activation. It is noteworthy that the effects of TRIM27-mediated activation of JNK/p38 pathways and inhibition of NF-κB pathway were more obvious during mycobacterial infection in the absence of PtpA (Fig. 3a,b), suggesting that PtpA might counteract the function of the TRIM27. Since Mtb PtpA was reported to be a tyrosine phosphatase and a variety of host substrates have been identified^14^,^16^,^20^,^31^, we thus sought to determine whether TRIM27 is a substrate of Mtb PtpA, and our data from *in vitro* dephosphorylation assay suggested an opposite result (Supplementary Fig. 3). In addition, the PtpA phosphatase inactive mutant (D126A) was also able to interact with the RING domain of TRIM27 (Supplementary Fig. 4), and the BCG strain expressing the PtpA D126A mutant had the same antagonizing effects as the WT BCG (Fig. 3a,b).

To further investigate the effects of TRIM27 on regulating the NF-κB and JNK/p38 pathways in macrophages, we transfected control siRNA or TRIM27 siRNA into U937 cells and infected with BCG stain for 0–48 h, followed by the phosphorylation level detection of IκBα, JNK, p38 and Erk. Immunoblot analysis data showed that knockdown of TRIM27 in U937 cells led to the suppression of JNK and p38 phosphorylation but increased IκBα phosphorylation compared with wild-type (WT) U937 cells, especially at 48 h post-infection. Erk pathway was not obviously regulated by TRIM27 in infected U937 cells (Fig. 3c). Collectively, these results indicate that TRIM27 regulates the NF-κB and JNK/p38 pathways during mycobacterial infection, which function could be antagonized by the secreted mycobacterial effector protein PtpA, independent of its phosphatase activity.

### Mtb PtpA antagonizes TRIM27-mediated cytokine production promotion and mycobacterial intracellular survival inhibition

**Figure 4:**
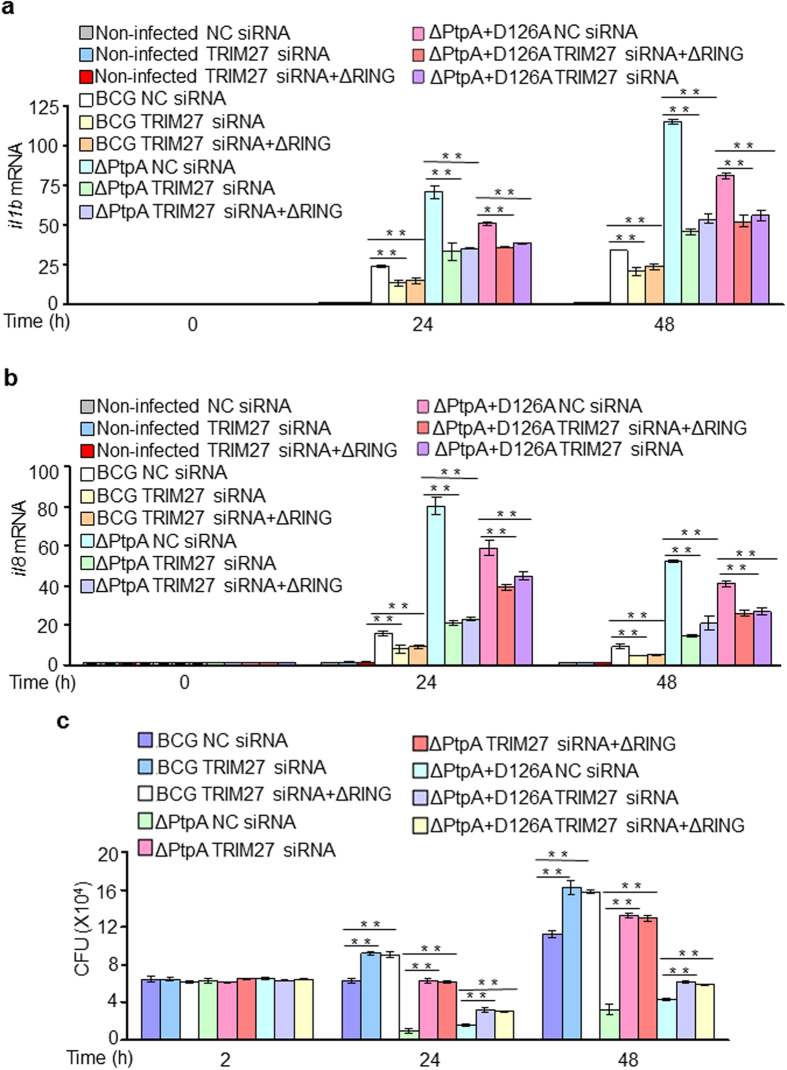
Mtb PtpA antagonizes TRIM27-promoted cytokine production and TRIM27-inhibited mycobacterial intracellular survival. (**a,b**) Quantitative PCR analysis of *il1b* mRNA (**a**) and *il8* mRNA (**b**) in U937 cells. Cells were transfected with NC siRNA or TRIM27 siRNA or TRIM27 siRNA complemented with TRIM27 (ΔRING), and then were infected with WT BCG or BCGΔPtpA or BCG (ΔPtpA+D126A) at a MOI of 10 for 0–48 h. Non-infected cells served as a control group. Analysis of each gene was normalized to GAPDH. (**c**) Survival of BCG in U937 cells treated as in (**a,b**). Data are shown as the means ± s.e.m. *P < 0.05 and **P < 0.01.

Cytokines, as the products of the immune signaling activation, are essential in host defense against invading pathogens. Our data indicated that TRIM27 potentiates anti-mycobacterial responses through mediating innate immune signaling pathways, which may lead to production of a number of cytokines to activate the immune system. To determine which cytokines are modulated by TRIM27 during mycobacterial infection, we treated WT U937 or TRIM27-knockdown (KD) U937 or TRIM27-KD U937 cells complemented with RING domain deleted TRIM27 (TRIM27 ΔRING) cells with BCG for 0–48 h, and analyzed the transcription level of *il1b* and *il8* by quantitative real-time PCR (Fig. 4a,b) and the protein level of IL-1β and IL8 secreted to the medium by Enzyme-linked immunosorbent assay (ELISA) (Supplementary Fig. 5a,b). Non-infected cells were used as a control. We found that knockdown of TRIM27 efficiently suppressed the production of IL-1β and IL8 in BCG-infected macrophages since 2 h post-infection, and the complementation with TRIM27 ΔRING exhibited the same suppression effects (Fig. 4a,b; Supplementary Fig. 5a,b). Thus, TRIM27 promotes the production of several cytokines (such as IL-1β and IL8) during mycobacterial infection, which depends on its RING domain. As with its antagonizing effects towards TRIM27-mediated regulation of the NF-κB and JNK/p38 pathways, PtpA also counteracted the TRIM27-promoted production of IL-1β and IL8 during mycobacterial infection in a phosphatase activity-independent manner (Fig. 4a,b; Supplementary Fig. 5a,b). Furthermore, TRIM27 could better inhibit the intracellular survival of mycobacteria in the absence of PtpA, and both WT BCG and the PtpA phosphatase-inactive mutant (D126A) strain could counteract this inhibitory effect, though the overall CFUs of the PtpA D126A mutant strain were much lower than that of the WT BCG (Fig. 4c). Together these data demonstrate that PtpA plays a critical role in counteracting the restriction effects of TRIM27 against mycobacterial infection in a phosphatase activity-independent manner.

### Mtb PtpA antagonizes TRIM27-promoted macrophage apoptosis during mycobacterial infection

**Figure 5:**
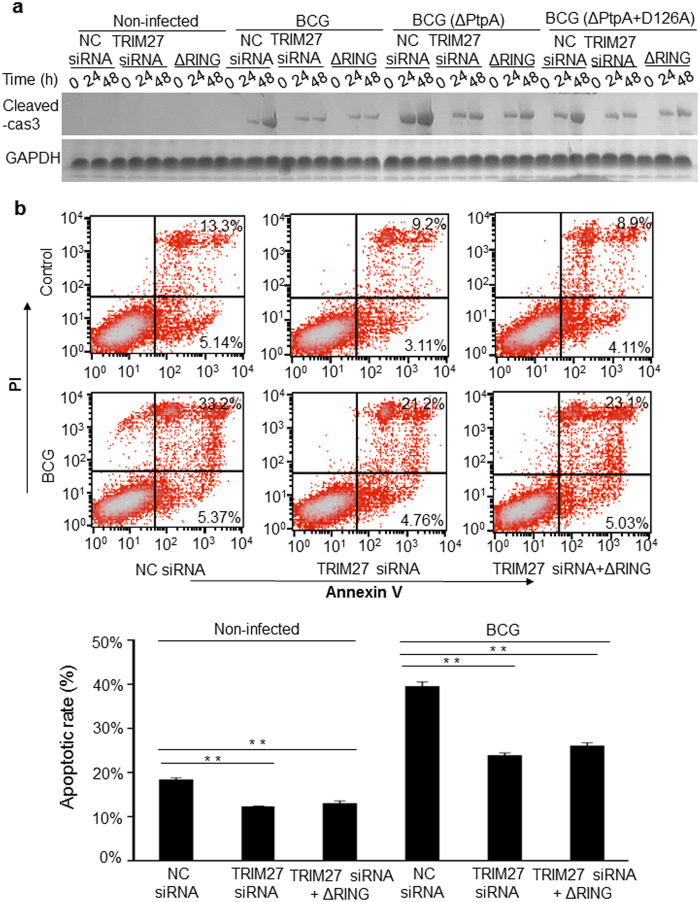
Mtb PtpA antagonizes TRIM27-promoted cell apoptosis. (**a**) Immunoblot analysis of cleaved caspase 3 in U937 cells. Cells were transfected with NC or TRIM27 siRNA or TRIM27 siRNA complemented with TRIM27 (ΔRING), and were then infected with WT BCG or BCGΔPtpA or BCG (ΔPtpA+D126A) stain at a MOI of 10 for 0–48 h. Non-infected cells served as a control group. GAPDH was used as a loading control. (**b**) U937 cells were treated as in a and were infected with BCG at a MOI of 10. After 24 h, the cells were stained with PI and annexin V, followed by flow cytometry analysis (top) and statistical analysis (bottom). Data are shown as the means ± s.e.m. *P < 0.05 and **P < 0.01.

In response to bacterial infection, programmed cell death, such as apoptosis, is induced as host innate immune responses to eliminate pathogens at the early stage of infection. Previous reports suggested that TRIM27 suppresses NF-κB activation^32^, and it can also ubiquitinate the ubiquitin-specific protease USP7, which deubiquitinates receptor-interacting protein1 (RIP1), leading to the promotion of cell apoptosis^28^. However, no evidence exists to indicate whether TRIM27 plays a role in the regulation of macrophage apoptosis during mycobacterial infection. Therefore, we performed immunoblot analysis to examine the level of cleaved caspase 3, a marker of apoptosis, in WT U937, TRIM27-KD U937 and TRIM27-KD U937 complemented with TRIM27 (ΔRING) cells infected with BCG for 0–48 h. Non-infected cells were used as a control. We observed a much higher level of cleaved caspase 3 in WT U937 cells than that in TRIM27-KD U937 cells or TRIM27 (ΔRING)-expressing U937 cells, especially at 48 h post-infection (Fig. 5a). Again, the effects of TRIM27-promoted cell apoptosis were more obvious during mycobacterial infection in the absence of PtpA, and the BCG strain expressing the PtpA D126A mutant had the same antagonizing effects as the WT BCG (Fig. 5a). Consistently, flow cytometry analysis data showed that the number of the early and late apoptosis cells significantly reduced in TRIM27-KD U937 cells and TRIM27 (ΔRING)-expressing U937 cells compared with that in WT U937 cells at 24 h post- infection (Fig. 5b). Thus, TRIM27 plays an important role in promoting macrophage apoptosis during mycobacterial infection, which function is also dependent on its RING domain and antagonized by Mtb PtpA in a phosphatase activity-independent manner.

### Global profiling of TRIM27-interacting proteins of Mtb

**Figure 6:**
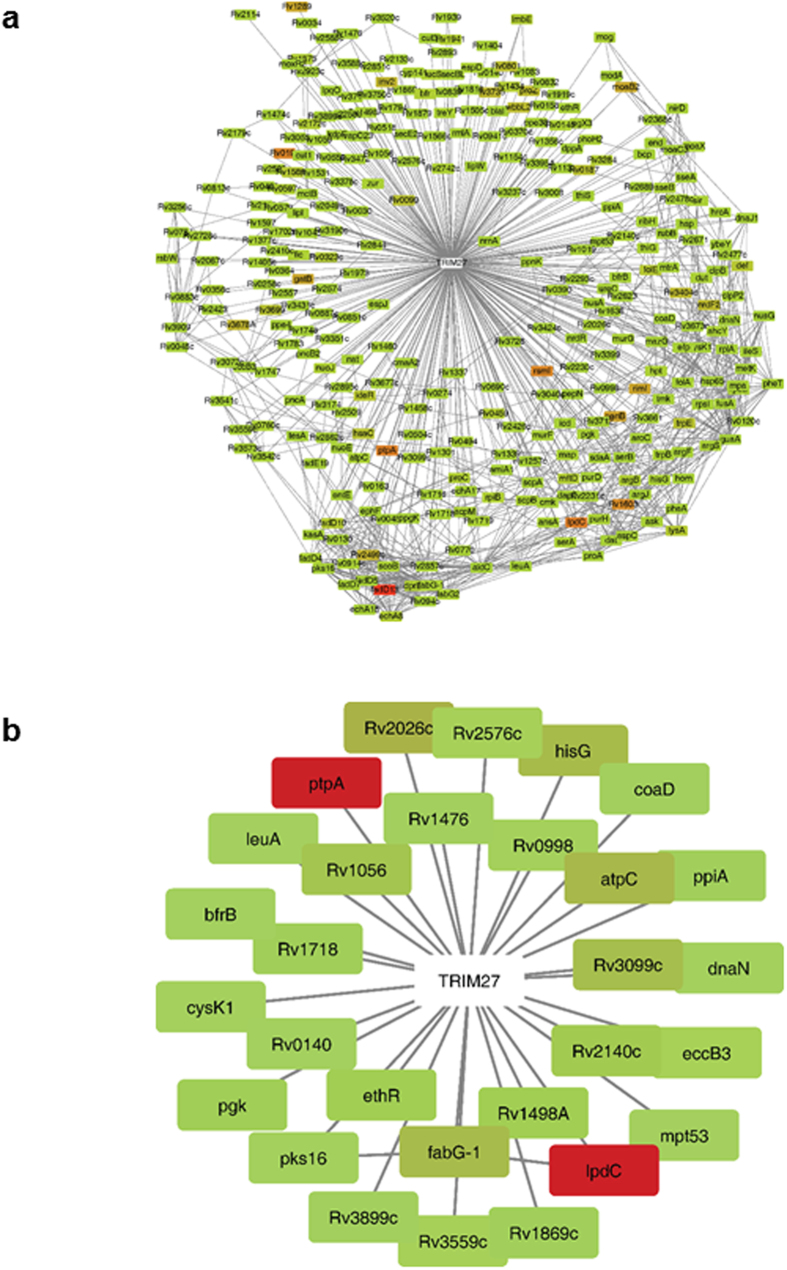
TRIM27-interacting Mtb proteins visualized by Cytoscape. (**a**) TRIM27-interacting total Mtb proteins visualized by Cytoscape with fold change data incorporated (red = high fold change; green = low fold change). (**b**) TRIM27-interacting secreted Mtb proteins visualized by Cytoscape with fold change data incorporated (red: large fold change; green: small fold change).

As described above, after infection of WT U937 cells, the CFUs of *M. smegmatis* markedly reduced and less than 10% bacteria survived at 48 h post-infection (Fig. 2c). In contrast, the CFUs of BCG began to increase since 8 h post-infection and reached its highest level at 48 h post-infection (Fig. 2b). This phenomenon, together with the observation that PtpA might antagonize the TRIM27-regulated immune-inflammatory responses and cell apoptosis during mycobacterial infection, prompted us to surmise that there might be certain effector proteins (which include but are not limited to PtpA) from Mtb which are able to interact with TRIM27 and antagonize its function during infection. We thus adopted the recently developed MTB Proteome Microarray to identify TRIM27-interacting Mtb proteins. The microarray contains more than 4,000 Mtb proteins. Purified GST and GST-TRIM27 were used to probe the microarray separately (Supplementary Fig. 6a). Using the stringent criteria described in Methods, we identified 321 candidate TRIM27-interacting proteins (Supplementary Dataset 1), 29 of which are known as secreted proteins (Supplementary Dataset 2). Six positive secreted proteins with the strongest interactions (calling score > 5) are shown in Supplementary Fig. 6b. All candidate TRIM27-interacting Mtb proteins and secreted proteins were analyzed respectively using STRING (Fig. 6a,b). The functional associations among these proteins were further analyzed using Cytoscape plugin ClueGO to decipher functionally grouped gene ontology (GO) and pathway annotation (Supplementary Fig. 7 and Supplementary Dataset 3). These TRIM27-interacting proteins were mainly involved in the metabolism (such as AtpC, HisG and FabG)^33^,^34^,^35^, stress reaction (such as Rv2026c) and intercellular survival (such as LpcC, PtpA, CysK1 and Pks16)^36^,^37^ of Mtb. As expected, Mtb PtpA was among the secreted proteins showing the strongest interaction with TRIM27 (Fig. 6b). These results suggest that TRIM27 functions as an important restriction factor regulating the intracellular survival and replication of mycobacteria, the functions of which might be counteracted by certain Mtb secreted effector proteins such as PtpA during mycobacterial infection.

## Discussion

Eukaryotic organisms have been exposed to pathogens for millions of years. This process has driven the development of immune responses against invading pathogens. Some proteins called “restriction factors” are evolved to provide considerable resistance to pathogen infection. Previous studies have identified many virus restriction factors, such as SAMHD1 and TRIM family members^8^,^12^,^38^, but few studies have reported restriction factors for bacteria pathogens, especially for Mtb, a leading cause of death in a global scale. In this study, we reveal that TRIM27 functions as a potential restriction factor which suppresses the intracellular survival of mycobacteria by enhancing host immune-inflammatory responses mediated by JNK/p38 pathways in an E3 ubiquitin ligase activity-dependent manner. Though TRIM proteins have been demonstrated as the key components of host antiviral innate immunity^10^, this is the first report demonstrating that TRIM27, another member from the TRIM protein family, could be added to the list of the increasing host restriction factors which plays important immune-regulatory roles during mycobacterial infection, and it might restrict other bacterial and viral pathogens as well.

To resist clearance by host immune defenses, pathogens have also evolved numerous countermeasures to escape host immunity. One of the most important strategies adopted by the bacterial pathogens is to produce various effector proteins to perturb host immune responses by targeting the key components of the innate immune system^1^. Mtb is known to be capable of persisting in host macrophage cells for a long time by producing various effector proteins to interfere with host signaling pathways. The main effector proteins of Mtb include 11 eukaryotic-like serine/threonine protein kinases (PknA-PknL), 2 protein tyrosine phosphatases (PtpA and PtpB), and the newly identified protein tyrosine kinase (PtkA), etc.^39^. We previously reported that Mtb tyrosine phosphatase PtpA, an important secreted effector protein required for intracellular survival of Mtb, inhibits innate immune signaling pathways and phagosome maturation by coopting host ubiquitin system^16^. In this study, we revealed novel immune-regulatory mechanisms of Mtb PtpA. PtpA not only can directly dephosphorylate phosphor-JNK and p38 as we previously reported, but also can interacts with TRIM27 and counteracts its restriction effects towards mycobacterial infection through regulating JNK/p38/NF-κB pathways and cell apoptosis. Since Mtb PtpA is delivered into macrophages during infection and its amino acid sequence is 37% identical to that of the human low-molecular-weight phosphotyrosine phosphatase HCPTPA, several previous studies have made efforts to identify the host substrates of Mtb PtpA. So far, several confirmed (such as VPS33B, GSK3a, JNK and p38) and some more potential substrates of PtpA have been identified^14^,^16^,^20^,^31^. In this study, we also sought to determine whether TRIM27 is a substrate of PtpA, and our data denied this possibility. Since our data indicated that the secreted PtpA interacts with TRIM27 in macrophages during mycobacterial infection, and both WT Mtb PtpA and its D126A mutant interact with the RING domain of TRIM27, we thus propose that PtpA blocks the E3 ubiquitin ligase activity of TRIM27 by binding competitively to its E3 ubiquitin activity-associated RING domain, thus leading to the antagonization of the TRIM27-mediated host innate immune signaling and cell apoptosis. Taken together, though the phosphatase activity of PtpA is critical in suppressing the activation of Jnk and p38 and the ensuing host innate immune responses^16^, it is not required for the PtpA-mediated antagonizing effects of TRIM27 activity during mycobacterial infection.

After infection by mycobacteria, the protein levels of TRIM27 in macrophages significantly increased since 2 h post-infection and reached its highest level at 8 h post-infection, followed by a sharp decrease in later time points. Concurrently, the CFUs decreased from 2 h post-infection of either BCG or *M. smegmatis*, but differences in the CFUs dynamics between BCG and *M. smegmati*s were observed during the later time points: while the CFUs of the *M. smegmati*s decreased consistently, the CFUs of the BCG increased after 24 h post-infection. These findings suggested that the protein levels of TRIM27 are highly regulated according to the existing CFUs of the pathogens to meet its function in restricting the intracellular survival of the pathogens. On the contrary, to promote their intracellular survival, certain intracellular pathogens such as the slow-growing and diseases-causing *Mycobacterium tuberculosis* complex (MTBC) members of mycobacteria such as BCG and Mtb are able to secrete effector proteins such as PtpA to antagonize the restriction effects of host factors such as TRIM27 at later time points post-infection. Therefore, our findings highlight the evolutionary dynamics of toggling between a restriction factor and its pathogen antagonist during mycobacterial infection.

In conclusion, our study demonstrated that on the one hand, TRIM27 functions as a potential restriction factor for mycobacteria; and on the other hand, pathogens such as BCG and Mtb have evolved to antagonize the restriction functions of TRIM27 through secreting immune-regulatory effector proteins such as PtpA. Our study could have a fundamental impact on promoting efforts in the development of novel anti-TB therapies based on pathogen-host interfaces (such as the TRIM27-PtpA interaction interfaces).

## Methods

### Bacterial strains, mammalian cell lines and antibodies

*E. coli* DH5α and BL21 were grown in flasks using LB medium. *M. smegmatis* mc^2^ 155 and *M. bovis* BCG (Pasteur) strains were grown in Middlebrook 7H9 broth (7H9) supplemented with 10% oleic acid–albumin–dextrose–catalase (OADC) and 0.05% Tween-80 (Sigma), or on Middlebrook 7H10 agar (BD) supplemented with 10% OADC. HEK293T (ATCC CRL-3216) and the human monocytic cell line U937 cells (ATCC CRL-1593.2) were obtained from the American Type Culture Collection (ATCC). HEK293T cells were maintained in Dulbecco’s modified Eagle’s medium (Gibco) with 10% fetal bovine serum (FBS), and U937 cells were maintained in RPMI 1640 medium with 10% FBS. For infection, U937 cells were cultured for 2 d in culture medium supplemented with 10 ng/ml of Phorbol 12-myristate 13-acetate (PMA, Sigma). The following antibodies were used in this study: anti-Myc (sc-40, Santa Cruz), anti-Flag (F3165, Sigma), anti-β-actin (A2228, Sigma), anti-phospho-Jnk (sc-81502, Santa Cruz), anti-phospho-IκBα (sc-101713, Santa Cruz), anti-phospho-p38 (9211S, Cell Signaling), anti-phospho-Erk (9100S, Cell Signaling), anti-phospho-tyrosine (Sigma). In Supplementary Table 1, we list detailed information on strains, plasmids and oligonucleotides used in this study.

### Immunoblot analysis and immunoprecipitation

For immunoblot analysis, cells were washed twice with ice-cold PBS, scraped, pelleted and lysed in the cell lysis buffer for Western and IP (P0013, Beyotime). After incubation for 10 min on ice, cell lysates were centrifuged at 14,000 rpm for 10 min at 4 °C. Protein concentration of lysates was determined by BCA protein assay kit (P0010, Beyotime) and the lysates were adjusted with cell lysis buffer. Proteins were separated by 8–15% SDS-PAGE transferred to PVDF membrane (Millipore). The blots were blocked for 1 h at room temperature with 5% non-fat dry milk in TBST (TBS and 0.05% Tween-20). Incubation with specific primary antibodies was performed in blocking buffer overnight at 4 °C. Goat anti-mouse IgG or goat anti-rabbit IgG conjugated to HRP was used as secondary antibody. The blots were developed by Immobilon Western Chemiluminescent HRP Substrate (WBKLS0500, Millipore) according to the manufacturer’s instructions. For immunoprecipitation, transfected cells were lysed in the Western and IP buffer (P0013, Beyotime). Cell lysates were incubated with Flag M2 beads (A2220, Sigma) or anti-Myc (9E10) beads (sc-40AC, Santa Cruz) and were analyzed by SDS-PAGE and blotted with indicated antibodies.

### RNA interference

U937 cells were transfected with 50 nM small interfering RNA with Lipofectamine™ RNAiMAX (13778, Invitrogen) according to the manufacturer’s instructions. Twelfth hours after the first transfection, cells were re-transfected as above, and the cells were incubated for a further 24 h before harvest for infection and analysis. The small interfering RNA sequences were as follows: TRIM27 siRNA, forward - ggagaaaatccaagaattauu, reverse - uaauucuuggauuuucuccuu; Luciferase siRNA (NC siRNA), forward - uucuccgaacgugucacguuu, reverse - acgugacacguucggagaauu.

### Real-time PCR

Total RNA was extracted from cells using TRIzol reagent (Invitrogen). The RNA was then used in a reverse transcription reaction using the RT-kit (R1012, Dongsheng Biotech). cDNA was then analyzed by quantitative PCR with KAPA SYBR FAST qPCR Kit (KAPA Biosystems) on ABI 7300 system (Applied Biosystems). Assays were performed in triplicates and three independent experiments were performed. Data are presented as mean values ± s.e.m. The following primer sequences (5′-3′) were used: *tnf*, forward - cctctctcttaatcagccctctg, reverse - gaggacctgggagtagatgag; *il1b*, forward - atgatggcttattacagtggcaa, reverse- gtcggagattcgtagctgga; *gapdh*, forward - ggagcgagatccctccaaaat, reverse - ggctgttgtcatacttctcatgg.

### ELISA quantification of cytokines

U937 cells (2 × 10^5^/well) were cultured in a 6-well plate for 1 d in culture medium supplemented with PMA. Levels of IL-1β in the collected medium were measured using ELISA quantification kit (KHC0011, Invitrogen) according to the manufacturer’s instructions. Assays were performed in triplicates and three independent experiments were performed. Data are presented as mean values ± s.e.m.

### Yeast two-hybrid assay

Yeast two-hybrid assay was performed using the Matchmaker Two-Hybrid System (Clontech) by following the manufacturer’s instructions. The mouse cDNA library Mate & Plate Library (CATALOG No. 630478; Clontech Laboratories, Inc.) was used to identify host interaction proteins of Mtb PtpA with yeast two-hybrid assay. Mtb PtpA gene was subcloned into the plasmid pGBKT7 as the bait plasmid. Saccharomyces cerevisiae AH109 cells were cotransduced with the bait plasmids and the prey plasmids by the lithium acetate method. To test the interactions between proteins, the transformants were streaked onto low-stringency (lacking leucine and tryptophan) and high-stringency (lacking adenine, histidine, leucine and tryptophan) selection plates.

### Luciferase reporter assay

Luciferase reporter assay was performed using the Promega luciferase reporter system. To measure NF-κB activation, HEK293T cells were co-transfected with 1 μg of pNF-κB-Luc and 50 ng of pRL-TK with or without 1 μg of TRIM27 plasmid. 24 h later, cells were treated with 40 ng/ml of TNF (Invitrogen) for 6 h. For JNK and p38 pathways activation, HEK293T cells were co-transfected with 0.3 μg of pFA-cJun, 0.9 μg of Gal4-luc, 50 ng of pRL-TK and 0.5 μg of RacL61 with or without 1 μg of TRIM27 plasmid. The fold induction was calculated as (relative light units stimulated)/(relative light units unstimulated).

### Immunouorescence confocal microscopy

U937 cells were seeded at 4 × 10^6^ cells/well in a 6-well plate and differentiated with 10 ng/ml PMA. After 24 h, cells were infected with BCG for 4 h at a MOI of 20 and washed three times, fixed in 4% paraformaldehyde, permeabilized with 0.5% Triton X-100 and blocked with BSA for 1 h, and labeled with antibodies against PtpA (prepared as described previously^16^) and TRIM27 (sc-47513, Santa cruz). Bacteria were stained with Alexa 350 carboxylic acid succinimidyl eater (A101678, Invitrogen). Confocal images were taken with a Leica SP8 confocal system and were processed with the Leica LAS AF Lite program.

### Annexin V and propidium iodide (PI) assay using flow cytometry

U937 cells were differentiated with 10 ng/ml PMA. After 24 h, the cells were washed once and cultured in fresh RPMI-1640 medium for an additional 8 h before infection. The U937 cells were infected with mycobacterial strains at a multiplicity of infection (MOI) of 10:1. After 24 h incubation, the media were discarded, and washed twice with PBS, and resuspended in 500 μl of 1×binding buffer. Annexin V-propidium iodide (PI) was then added in accordance with the Annexin V-PI Apoptosis Detection Kit (C1062, Beyotime) protocol. Flow cytometry (BD FACSCalibur) was used for the quantification of apoptotic cells.

### MTB Proteome Microarray assay

A total of 4262 proteins from MTB strains were spotted in duplicate on polymer slides (microarray-TB-12, BCBio). MTB proteome microarrays were blocked for 1 h at room temperature with shaking in blocking buffer (3% BSA, 1 × TBST, and 0.1% Tween 20 [pH 7.4]). To identify TRIM27-interacting proteins, we probed the MTB proteome microarray with a biotinylated-TRIM27 (0.05 mg/ml). Biotinylated-BSA (0.05 mg/ml) was used as negative controls. After incubating overnight at 4 °C with shaking, arrays were washed three times with shaking in 1 × TBST and then probed with 3 ml Cy3-strepavadin (1 mg/mL in 1 × TBST buffer; Sigma-Aldrich) for 1 h at room temperature with shaking. After washing three times in 1 × TBST and once in ddH_2_O, arrays were dried and then scanned. The signal to noise ratio (SNR) of each protein was averaged for the two duplicated spots on each microarray incubated with GST (SNR^−^) or GST-TRIM27 (SNR^+^). To identify which proteins had significantly higher SNRs in the microarray incubated with GST-TRIM27 than in the control microarray, calling score was defined as SNR^+^/SNR^−^ and calculated for all the proteins. Cutoff values were set as calling score > 2 and SNR > 3 for TRIM27-interacting proteins.

### Analysis of protein interaction networks

The protein interaction networks were analyzed using STRING 10^40^ and visualized using Cytoscape 3.3.0^41^. The protein association network constructed by STRING was further analyzed using Cytoscape plugin ClueGO to decipher functionally grouped gene ontology and pathway annotation. GO enrichment of the network data was carried out to detect significantly overrepresented GO in terms of their associated biological processes components. The P value was calculated with a two-sided minimal-likely-hood test on the hypergeometric distribution. The degree of connectivity between GO terms was calculated using kappa statistics. The calculated kappa score of ≥0.3 was used for defining functional groups.

### Statistical analysis

The data in the graphs are expressed as the mean ± s.e.m. Two-tailed unpaired t-test with Welch’s correction was used for statistical analysis. Each value of *P < 0.05 or **P < 0.01 was considered to be statistically significant.

## Additional Information

**How to cite this article**: Wang, J. *et al*. The ubiquitin ligase TRIM27 functions as a host restriction factor antagonized by *Mycobacterium tuberculosis* PtpA during mycobacterial infection. *Sci. Rep.* **6**, 34827; doi: 10.1038/srep34827 (2016).

## Supplementary Material

Supplementary Information

Supplementary Dataset 1

Supplementary Dataset 2

Supplementary Dataset 3
