# Supplementary material for: The ubiquitin ligase TRIM27 functions as a host restriction factor antagonized by Mycobacterium tuberculosis PtpA during mycobacterial infection
Source: Sci Rep. 2016 Oct 4;6:34827. doi: 10.1038/srep34827 (PMC5048167; doi:10.1038/srep34827)
Supplement: Supplementary Information [file srep34827-s1.doc]

**Supplementary information**

**The ubiquitin ligase TRIM27 functions as a host restriction factor antagonized by *Mycobacterium tuberculosis* PtpA during mycobacterial infection**

**Jing Wang^1^, Jade L. L. Teng^3^, Dongdong Zhao^1, 2^, Pupu Ge^1^, Bingxi Li^1^, Patrick C. Y. Woo^3^ & Cui Hua Liu^1, 2, *^**

^1^ CAS key Laboratory of Pathogenic Microbiology and Immunology, Institute of Microbiology, Chinese Academy of Sciences, Beijing 100101, China

^2^ Savaid Medical School, University of Chinese Academy of Sciences, Beijing 101408, China

^3^ Department of Microbiology, The University of Hong Kong, Hong Kong, China

^*^ Correspondence and requests for materials should be addressed to C.H.L. (email: [liucuihua@im.ac.cn](mailto:liucuihua@im.ac.cn))

**
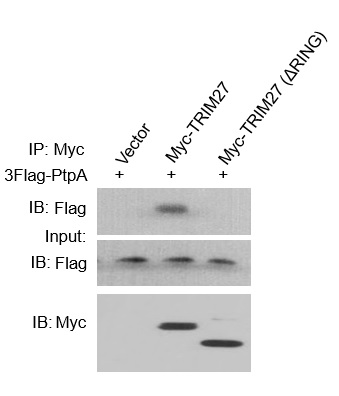
**

**Supplementary Figure 1: Deletion of RING domain in TRIM27 disrupts the interaction between TRIM27 and *Mycobacterium tuberculosis* (Mtb) PtpA.** Immunoblot analysis of proteins immunoprecipitated with anti-Myc from lysates of HEK293T cells cotransfected with vectors encoding Flag-tagged Mtb PtpA and Myc-tagged full-length (FL) TRIM27 or RING domain-deleted TRIM27.


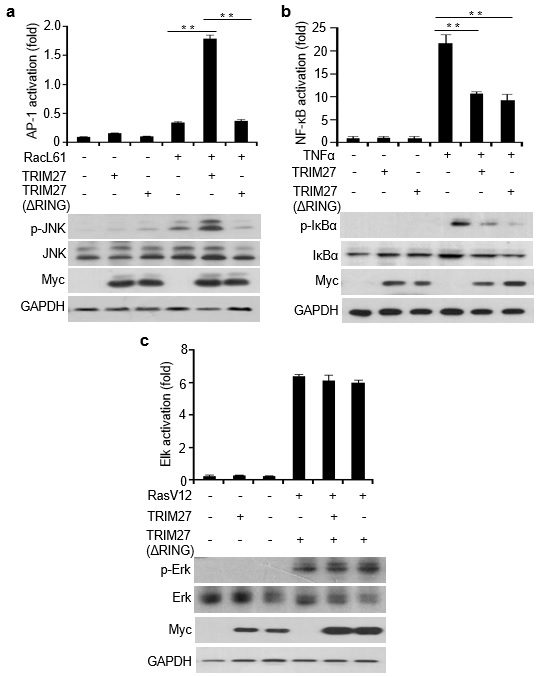


**Supplementary Figure 2: TRIM27 promotes the activation of JNK and p38 pathways and suppresses NF-κB activation in HEK293T cells.** Luciferase assay (top) and immunoblot analysis (bottom) of AP-1 **(a),** NF-κB **(b)** and Elk **(c)** activation by overexpression of Myc-tagged TRIM27 in HEK293T cells. Cells were treated with TNFα for induction of NF-κB pathway and transfected with constitutively active RacL61 or RasV12 for activation of JNK/p38 or Erk pathway, respectively.


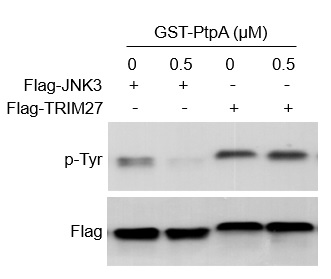


**Supplementary Figure 3: TRIM27 is not the substrate of Mtb PtpA.** JNK3 and TRIM27 were obtained by immunoprecipitation from cell lysates cotransfected with vectors encoding Flag-JNK3 or Flag-TRIM27 using anti-Flag M2 beads and were titrated with 0 or 0.5 μM of purified Mtb PtpA at 30 °C for 1 h, followed by immunoblot analysis of phospho-tyrosine and Flag-tagged proteins (loading control).


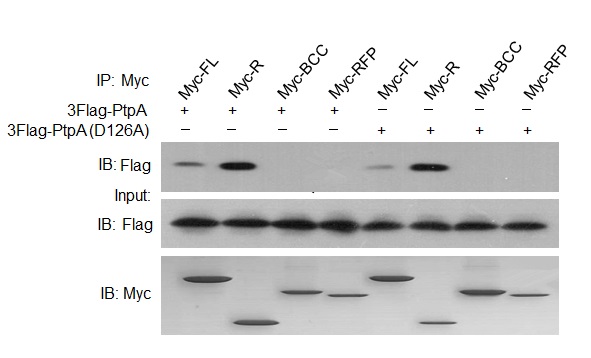


**Supplementary Figure 4: Mtb PtpA phosphatase-inactive mutant (D126A) interacts with the RING domain of TRIM27.** Immunoblot analysis of proteins immunoprecipitated with anti-Myc from lysates of HEK293T cells cotransfected with vectors encoding Flag-tagged PtpA (or its D126A mutant form) and Myc-tagged full-length (FL) TRIM27 or its truncated forms (R, RING; BCC, B-Box region and coiled-coil region; RFP, RFP region).

###

###
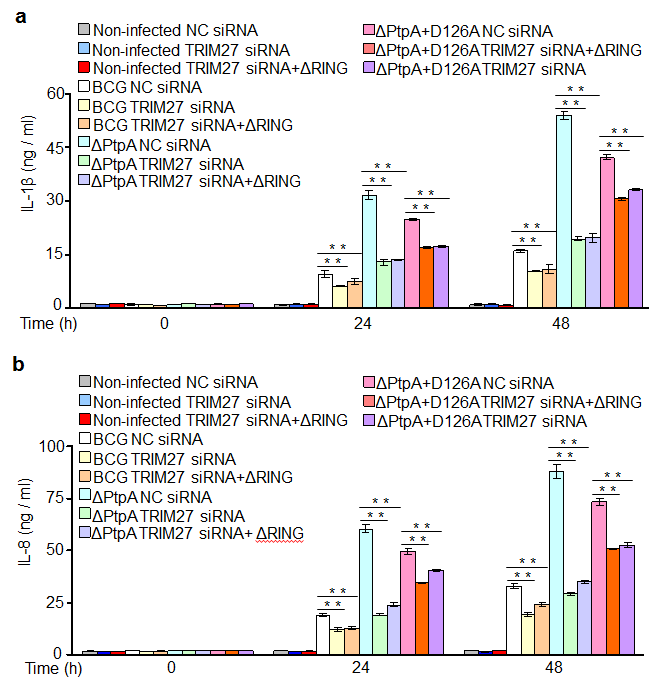


**Supplementary Figure 5: Mtb PtpA antagonizes TRIM27-promoted production of IL-1β and IL-8 in a phosphatase activity-independent manner during mycobacterial infection.** Enzyme-linked immunosorbent assay (ELISA) of IL-1β **(a)** and IL-8 (b) in the medium of U937 cells transfected with luciferase siRNA (NC siRNA) or TRIM27 siRNA or transfected with TRIM27 siRNA complemented with RING domain deleted TRIM27 and infected with wild-type (WT) BCG or BCG (ΔPtpA) or BCG (ΔPtpA) complemented with PtpA phosphatase inactive mutant (ΔPtpA + D126A) at a MOI of 10 for 0-48 h. Non-infected cells served as a control group. Data are shown as the means ± s.e.m.; **P* < 0.05 and ** *P* < 0.01.


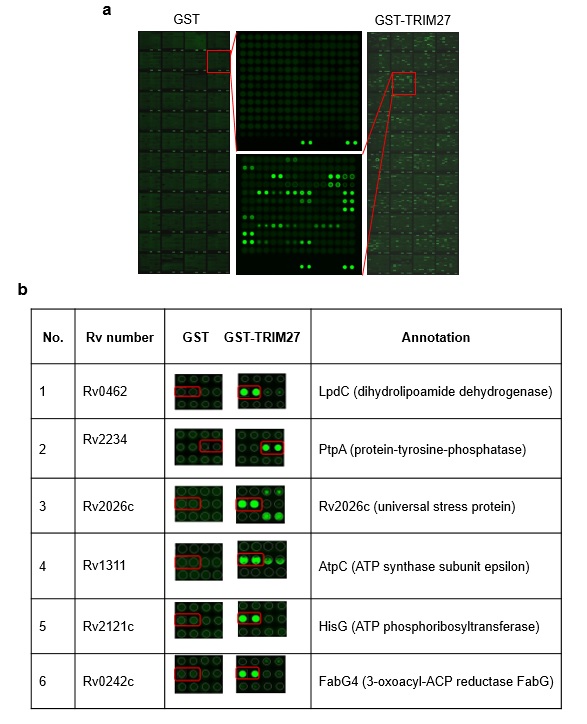


**Supplementary Figure 6: Identification of TRIM27-interacting proteins from Mtb. (a)** More than 4,000 proteins from *M. tuberculosis* strain H37Rv were spotted in duplicate on polymer slides. Positive spots were determined with the Mtb proteome microarray by probed with biotinylated GST or biotinylated GST-tagged TRIM27 protein, respectively. Outlined areas are enlarged in the middle. **(b)** Cutoff values were set as calling score > 2 and 321 positive spots were identified in this array. Only 6 positive TRIM27-interacting secreted proteins with calling score > 5 were listed in the table. 1-6: TRIM27 interacting-Mtb secreted proteins with calling score > 5. The calling scores are shown in increasing order from top to bottom.


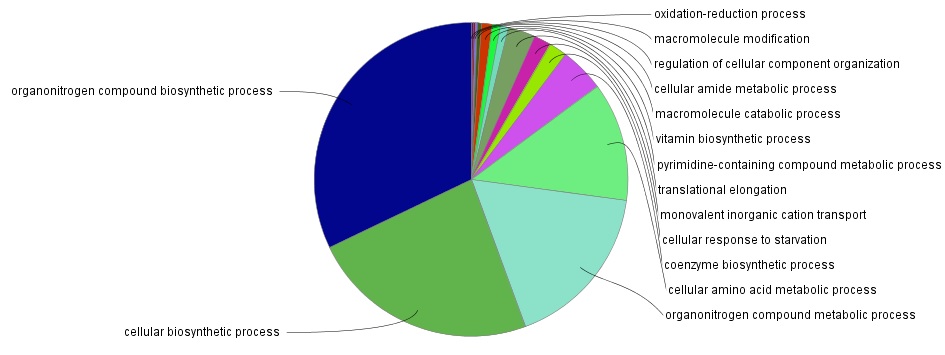


### Supplementary Figure 7: The overview chart presenting functional groups for TRIM27-interacting Mtb proteins. The name of the group was given by the group leading term, representing the most significant term in the group. The group sections correlated with the number of the terms included in group.

**Supplementary Table 1 plasmids, Bacterial strains and oligonucleotides used in this study.**

| **Name** | **Description** | **Reference** |
| --- | --- | --- |
| **Plasmids** | | |
| pGADT7 | For expression of proteins fused to a GAL4 activation domain (AD) | F. Shao |
| pGBKT7 | For expression of  proteins fused to amino acids 1-147 of the GAL4 DNA binding domain (DNA-BD) | F. Shao |
| pGADT7-TRIM27 | For expression of AD-TRIM27 in the yeast | This study |
| pGADT7-TAK1 | For expression of AD-TAK1 in the yeast | F. Shao |
| pGBKT7-PtpA | For expression of BD-PtpA in the yeast | This study |
| pGBKT7-TAB2 | For expression of BD-TAB2 in the yeast | F. Shao |
| p3xFlag-CMV14 | CMV promoter, for mammalian expression, 3xFlag tag, Amp^R^ | Sigma |
| p3xFlag-CMV14-PtpA | For expression of Flag-PtpA in mammalian cells | This study |
| p3xFlag-CMV14-PtpA (D126A) | For site-directed mutagenesis of PtpA | This study |
| p3xFlag-CMV14-TRIM27 | For expression of Flag-TRIM27 in mammalian cells | This study |
| p3xFlag-CMV14-JNK3 | For expression of Flag-JNK3 in mammalian cells | This study |
| pcDNA6A | T7 promoter, for mammalian expression, Myc tag, Amp^R^ | Invitrogen |
| pcDNA6A-TRIM27 | For expression of Myc-TRIM27 in mammalian cells | This study |
| pcDNA6A-TRIM27 (RING) | For expression of Myc-tagged RING domian of TRIM27 in mammalian cells | This study |
| pcDNA6A-TRIM27 (BCC) | For expression of Myc-tagged B-Box and Coiled-coil domain of TRIM27 in mammalian cells | This study |
| pcDNA6A-TRIM27 (RFP) | For expression of Myc-tagged RFP domian of TRIM27 in mammalian cells | This study |
| pcDNA6A-TRIM27 (ΔRING) | For expression of Myc-tagged RING domain-deleted TRIM27 in mammalian cells | This study |
| pET30a | T7 promoter, for bacterial expression, 6xHis tag, Kan^R^ | Novagen |
| pET30a-TRIM27 | For expression of recombinant protein His_6_-TRIM27 | This study |
| pGEX-6P-1 | *tac* promoter, for bacterial expression, GST tag,Amp^R^ | GE Healthcare |
| pGEX-6P-1-PtpA | For expression of recombinant protein GST-PtpA | This study |
| pcDNA3-RacL61 | For expression of constitutively activated Rac in mammalian cells | F. Shao |
| pcDNA3-RasV12 | For expression of constitutively activated Ras in mammalian cells | F. Shao |
| pNF-κB-luc | Used in dual-luciferase assay for NF-κB pathway | F. Shao |
| pRL-TK | Used in dual-luciferase assay for NF-κB and MAPK pathways | F. Shao |
| pGal4-luc | Used in dual-luciferase assay for MAPK pathway | F. Shao |
| pFA-cJun | Used in dual-luciferase assay for MAPK pathway | F. Shao |
| pGal4-Elk | Used in dual-luciferase assay for Erk pathway | F. Shao |
| **Strains** | | |
| *E. coli* DH5α | F^-^ φ80*lac*ZΔM15 Δ (*lac*ZYA-*arg*F) U169 *rec*A1 *end*A1 *hsd*R17 (rk-, mk+) *pho*A *sup*E44 λ^-^ *thi*^-^1 *gyr*A96 *rel*A1 | Invitrogen |
| *E.coli* BL21 (DE3) | F^-^ *ompT hsdS*_B_ (r_B_^-^ m_B_^-^) *gal dcm* (DE3) | Novagen |
| *M. bovis* BCG | Pasteur | ATCC 35734 |
| BCG△PtpA | BCG strain with deletion of PtpA | This study |
| △PtpA+PtpA (D126A) | BCG △PtpA strain complemented with PtpA (D126A) | This study |
| *M.smegmatis* | mc^2^155 | ATCC 700084 |
| **Oligonucleotides (5’-3’)** | | |
| pGADT7-TRIM27-F | TTAGAATTCATGGCCTCCGGGAGTGTG | This study |
| pGADT7-TRIM27-R | CTTGTCGACAGGGGAGGTCTCCATG | This study |
| pGBKT7-PtpA-F | CGAATTCGTGTCTGATCCGCTGCACGT | This study |
| pGBKT7-PtpA-R | GCGGATCCTCAACTCGGTCCGTTCCGCGCG | This study |
| p3xFlag-CMV14-TRIM27-F | TTAGAATTCATGGCCTCCGGGAGTGTG | This study |
| p3xFlag-CMV14-TRIM27-R | CGGGATCCAGGGGAGGTCTCCATGGAA | This study |
| p3xFlag-CMV14-JNK3-F | CGGGATCCATGAGCCTCCATTTCTTATAC | This study |
| p3xFlag-CMV14-JNK3-R | CCGCTCGAGTCACTGCTGCACCTGTGCTG | This study |
| p3xFlag-CMV14-PtpA-F | CCCAAGCTTGTGTCTGATCCGCTGCACGTC | This study |
| p3xFlag-CMV14-PtpA-R | GCTCTAGAACTCGGTCCGTTCCGCGCGAGACGT | This study |
| pGEX-6P-1-PtpA-F | TCTGAATTCGTGTCTGATCCGCTGCACGT | This study |
| pGEX-6P-1-PtpA-R | CCGCTCGAGTCAGTCGTGCAGGCCGGGCAGGGC | This study |
| pcDNA6A-TRIM27-F | TTAGAATTCATGGCCTCCGGGAGTGTG | This study |
| pcDNA6A-TRIM27-R | CTTGTCGACAGGGGAGGTCTCCATG | This study |
| pcDNA6A-TRIM27 (RING)-F | TTAGAATTCATGGCCTCCGGGAGTGTG | This study |
| pcDNA6A-TRIM27 (RING)-R | CATGTCGACCTTCAGGGGCTCGCGGTGCTTCTCGC | This study |
| pcDNA6A-TRIM27 (BCC)-F | TTAGAATTCATGCTGTACTGCGAGGAGGACCAGA | This study |
| pcDNA6A-TRIM27 (BCC)-R | CATGTCGACGATCAGGCTGGGGTA | This study |
| pcDNA6A-TRIM27 (RFP)-F | TCAGAATTCATGTCAGTGGACGTGACTC | This study |
| pcDNA6A-TRIM27 (RFP)-R | CTTGTCGACAGGGGAGGTCTCCATG | This study |
| pcDNA6A-TRIM27 (ΔRING)-F | TTAGAATTCATGCTGTACTGCGAGGAGGACCAGA | This study |
| pcDNA6A-TRIM27 (ΔRING)-R | CTTGTCGACAGGGGAGGTCTCCATG | This study |
| pET28a-TRIM27-F | CGGGATCCATGGCCTCCGGGAGTGTGGCC | This study |
| pET28a-TRIM27-R | CGAATTCTCAAGGGGAGGTCTCCATGGAA | This study |
| NC siRNA | UUCUCCGAACGUGUCACGUUU | This study |
| TRIM27 siRNA | ggagaaaauccaagaauuauu | This study |
| Il8-QRT-F | CTGGCCGTGGCTCTCTTG | This study |
| Il8-QRT-R | CTTGGCAAAACTGCACCTTCA | This study |
| Il1b-QRT-F | ATGATGGCTTATTACAGTGGCAA | This study |
| Il1b-QRT-R | GTCGGAGATTCGTAGCTGGA | This study |
| Gapdh-QRT-F | GGAGCGAGATCCCTCCAAAAT | This study |
| Gapdh-QRT-F | GGCTGTTGTCATACTTCTCATGG | This study |

**Supplementary Dataset 1**: The list of the 321 candidate TRIM27-interacting Mtb proteins identified with the MTB Proteome Microarray. Cutoff values were set as calling score >2 and SNR >3.

**Supplementary Dataset 2**: The list of the 29 candidate TRIM27-interacting Mtb secreted proteins identified with the MTB Proteome Microarray. Cutoff values were set as calling score >2 and SNR >3.

**Supplementary Dataset 3**: The detailed ClueGO results and the corresponding histogram with GO terms. The chart presents the specific GO terms for all 321 TRIM27-interacting Mtb proteins. The bars represent the number of the Mtb proteins found to be associated with the GO term.
